# Human Angiotensin I-Converting Enzyme Produced by Different Cells: Classification of the SERS Spectra with Linear Discriminant Analysis

**DOI:** 10.3390/biomedicines10061389

**Published:** 2022-06-12

**Authors:** Irina Boginskaya, Robert Safiullin, Victoria Tikhomirova, Olga Kryukova, Natalia Nechaeva, Naida Bulaeva, Elena Golukhova, Ilya Ryzhikov, Olga Kost, Konstantin Afanasev, Ilya Kurochkin

**Affiliations:** 1 Institute for Theoretical and Applied Electromagnetics RAS, 125412 Moscow, Russia; safiullin.rr@phystech.edu (R.S.); nanocom@yandex.ru (I.R.); kavacuum@mail.ru (K.A.); 2 Bakulev Scientific Center for Cardiovascular Surgery, Cardiology Department, 121552 Moscow, Russia; naida_bulaeva@yahoo.com (N.B.); egolukhova@yahoo.com (E.G.); 3 Moscow Institute of Physics and Technology, 141700 Dolgoprudny, Russia; 4 Faculty of Chemistry, M.V. Lomonosov Moscow State University, 119991 Moscow, Russia; vetikhomirova@gmail.com (V.T.); so.b11onde@gmail.com (O.K.); kost-o@mail.ru (O.K.); inkurochkin@gmail.com (I.K.); 5 Emanuel Institute of Biochemical Physics RAS, 119334 Moscow, Russia; nechaeva.n.l@yandex.ru; 6 FMN Laboratory, Bauman Moscow State Technical University, 105005 Moscow, Russia

**Keywords:** angiotensin I-converting enzyme, SERS, nanostructured surface, full spectra assignment, linear discriminant analysis, tissue-specificity, glycosylation

## Abstract

Angiotensin I-converting enzyme (ACE) is a peptidase widely presented in human tissues and biological fluids. ACE is a glycoprotein containing 17 potential N-glycosylation sites which can be glycosylated in different ways due to post-translational modification of the protein in different cells. For the first time, surface-enhanced Raman scattering (SERS) spectra of human ACE from lungs, mainly produced by endothelial cells, ACE from heart, produced by endothelial heart cells and miofibroblasts, and ACE from seminal fluid, produced by epithelial cells, have been compared with full assignment. The ability to separate ACEs’ SERS spectra was demonstrated using the linear discriminant analysis (LDA) method with high accuracy. The intervals in the spectra with maximum contributions of the spectral features were determined and their contribution to the spectrum of each separate ACE was evaluated. Near 25 spectral features forming three intervals were enough for successful separation of the spectra of different ACEs. However, more spectral information could be obtained from analysis of 50 spectral features. Band assignment showed that several features did not correlate with band assignments to amino acids or peptides, which indicated the carbohydrate contribution to the final spectra. Analysis of SERS spectra could be beneficial for the detection of tissue-specific ACEs.

## 1. Introduction

Raman spectroscopy is a common and well developed technical analytical method for biomedical applications [1,2]. However, this method has a limitation due to the low quantum yield, especially in the case of proteins. The discovery of the surface-enhanced Raman scattering (SERS) effect helped to increase the sensitivity and removed this limitation [3] through the use of nanostructured substrates. The development of the theory of the method and technologies for the formation of SERS substrates has led to the appearance of a number of substrates of a different nature [4,5,6,7,8,9,10,11,12,13,14]. The SERS method has been successfully applied for the study of serum albumin, serum glycated albumin, myoglobin, butyrylcholinesterase, and angiotensin-converting enzyme from seminal fluid [8,9,11,15,16,17], thus demonstrating perspectives of the method for analyzing a wide range of proteins. It should be especially noted that SERS can be applied for glycated human albumin biosensing [8]. Glycation is usually caused by an excess of sugar in the blood due to diabetes. The SERS method allowed discrimination of glycated and non-glycated albumin both in buffer and in the blood plasma.

Glycosylation is one of the most common of over 300 known post-translational modifications. For many proteins, glycosylation is an essential step in the synthesis, playing important roles in various biological processes, such as the regulation of protein folding and sorting, cell proliferation and differentiation, cell–cell recognition, protein–protein communication, adhesion, migration, and immune responses [18]. The multifaceted process of glycan synthesis is influenced by a large number of factors, including the compartmentalization of glycosyltransferases, the supply and transport of sugars and sugar nucleotides. These processes can change during the development of various pathologies, e.g., cancer, which affects the final structure of glycans [19]. These aberrant glycans can serve as biomarkers [20]. Even in healthy tissues, the pattern of glycosylation of the same protein can vary greatly depending on the type of cell in which the protein was synthesized. This feature can help to identify the origin of a protein [21,22,23,24].

To find out whether different glycosylation of the same protein could affect SERS spectra, we have chosen angiotensin I-converting enzyme (ACE, EC 3.4.15.1, CD143) as a model protein. ACE is a highly glycosylated type I protein expressed on the surface of endothelial, epithelial, neuroepithelial cells, as well as cells of the immune system (macrophages and dendritic cells) [25,26]. ACE is also present in the biological fluids of the organism, including blood, as a soluble form which originates by proteolytic cleavage of juxtamembrane sequence of the protein and loss of its transmembrane anchor [25]. The level of ACE in the blood can serve as a marker of some pathologies [27,28,29,30].

ACE produced in different cells is coded by the same gene [26]. However, enzymatic and immunological ACE properties can vary due to different glycosylation [31,32,33,34]. Human ACE contains 17 potential N-glycosylation sites, 10 of which are located on the N domain of the enzyme, and seven on the C domain [35]. However, the exact position of really occupied sites, as well the structure of glycans, is only partly characterized. Nevertheless, it was shown that ACE produced in various cells may differ in the number of actually glycosylated sites and in the structure of oligosaccharide chains [32,34,36,37,38]. Thus, ACE from human seminal fluid was shown to contain seven oligosaccharide chains, five of which were complex-type glycans while two appeared to be mannose-type [37]. The mass spectrometry of tryptic hydrolyzates of ACEs isolated from different human organs made it possible to reveal several N-glycosylation sites which are really occupied by glycans, as well as to demonstrate the presence of different glycan structures in different ACEs [32,34]. Such variability in the pattern of glycosylation of ACE from different tissues affects the surface structure of the ACE globule and, therefore, the pattern of ACE recognition by monoclonal antibodies to different epitopes on ACE surface [32,33,34,39].

We considered Raman spectroscopy as a promising method capable of distinguishing differently glycosylated ACEs produced in different cells. For this purpose, we used machine learning methods. Machine learning and statistical analysis methods can be adapted (trained) for many tasks, including spectroscopy. In Lussier’s work [40], the authors review the classic models used in mass, NMR, and Raman spectroscopy, as well as the problems solved with their help. Studies have demonstrated that a SERS-based detection platform can discriminate bacteria species using linear discriminant analysis (LDA) [41]. The spectra of different proteins were successfully separated using basic machine learning models [42]. A similar approach was further applied for the classification and interpretation of Raman spectra of proteins [43]. All of them show good performance in quantitative and discriminant analysis of organic substances. It is also worth mentioning that the overall quality of the analysis directly depends on the data and methods of its preprocessing. Common spectra preprocessing techniques are applicable [44] while an artificial method of increasing the sample size could be more advanced [45].

Classification methods are widely used for separation [46] and feature importance search [47] for further interpretation of these values. Specifically, LDA allows us to present data in an optimal low-dimensional space, where studied samples can be efficiently separated. Therefore, it is a powerful tool for multicollinear analysis of multidimensional data such as spectroscopy.

In this work, we obtained SERS spectra of three purified ACEs: ACE from lungs, mainly produced by endothelial cells of lung capillaries, ACE from heart, produced by endothelial heart cells and, probably, by miofibroblasts, and ACE from seminal fluid, produced by epithelial cells of prostate and epididymis. These SERS spectra of three different ACE types were distinguished using linear discriminant analysis.

## 2. Materials and Methods

### 2.1. ACE Isolation from Different Sources

The work was carried out in accordance with The Code of Ethics of World Medical Association (Declaration of Helsinki) and was approved by the Institutional Review Boards of the Bakulev Center of Cardiovascular Surgery, and the N.A. Lopatkin Research Institute of Urology and Interventional Radiology. None of the donors were from the vulnerable populations and all donors or next of kin provided written informed consent that was freely given. Seminal fluid, lung and heart tissues were used as sources of somatic two-domain ACEs. Lung and heart ACEs were purified from tissue homogenates using anion-exchange chromatography on DEAE-Toyopearl 650M and then lisinopril affinity chromatography as in [48,49]. Seminal fluid ACE was obtained by lisinopril affinity chromatography. Before landing on the SERS substrate, all ACE preparations were desalted by extensive washing with 1 μM ZnCl_2_ solution on 100 kDa filtration membranes (GE Healthcare, Sartorius Corp., Bohemia, NY, USA). Zinc salt was added to maintain the active conformation of ACE during desalination.

### 2.2. ACE Characterization

ACE activity in all samples was determined using fluorimetric assay with synthetic peptide Benzyloxycarbonyl-L-Phe-L-His-L-Leu (Bachem, Torrance, CA, USA) in 50 mM phosphate buffer, pH 7.5, containing 150 mM NaCl and 1 μM ZnCl_2_. Briefly, 20 μL aliquots of samples were added to 100 μL of 2,4 mM Benzyloxycarbonyl-Phe-His-Leu, incubated for the appropriate time at 37 °C and then the product of enzymatic hydrolysis, His-Leu, was quantified fluorimetrically via complexing with *o*-phtaldialdehyde [50].

Purified ACEs were proved to be homogeneous according to electrophoresis in 7.5% SDS-PAGE [51]. Protein concentrations were determined according to the modified Lowry method [52]. Purified ACE preparations were stored at −18 °C.

### 2.3. SERS Substrate Fabrication and Characterization

The substrates were formed using electron-beam evaporation in a URM 3.279.072 (Quartz Ltd., Kaliningrad, Russia) vacuum chamber according to the method described in [4]; 4N high-purity 3 mm granulated silver (99.99% Moscow special alloys processing plant, Russia) was used. Silver was deposited on glass slides (Heinz Herenz Medizinalbedarf GmbH, Hamburg, Germany) preliminarily purified with isopropyl alcohol (99.6% Sigma Aldrich, Burlington, MA, USA) and plasma on the residual atmosphere directly in the vacuum chamber at pressure 10^−3^ Torr, whereas operating pressure was 10^−6^ Torr. The residual atmosphere mainly consisted of nitrogen, which was used to ignite the plasma for additionally cleaning the substrates on which silver films were deposited.

As a result of sputtering, silver films 100 nm thick were obtained. The thickness control during application was carried out using optical transmission control at a wavelength of 545 nm.

### 2.4. SERS Measurements

The SERS spectra were measured using an Alpha 300 R confocal Raman spectrometer (WITec, Ulm, Germany), at excitation wavelength 785 nm, laser power 54 mW, acquisition time of 1 spectrum was 60 s, using a ZEISS 50X/0.8 Epiplan Neofluar lens. Aliquots of 5 μL of ACE solutions at an initial concentration of 0.3 μM were applied to the SERS substrate and dried in air. The spectra were measured from at least 60 points in the coffering region (Figure S1) of 5 drops for each type of ACE on one substrate. As a result, 197 samples were collected. Additionally, Raman spectra of ACEs were measured dried on glass slides (Heinz Herenz Medizinalbedarf GmbH, Hamburg, Germany) in the same manner as in SERS experiments and the spectra were measured in the same conditions.

### 2.5. Preprocessing Spectra for LDA

The following steps were used to preprocess 197 obtained spectra where each spectrum was taken with 1024 points. This number is determined by spectrometer resolution. First, the Raman shift range (300 to 1800 cm^−1^) was selected since this range contains all vibrational bands. Then, the baseline was corrected using a rubber-band correction and the outliers were eliminated according to the algorithm described in Supplementary (Figure S2). The reason for the existence of outliers is mainly cosmic rays. They are characterized by a high signal-to-noise ratio, exceeding this ratio at the same frequency. After that, each spectrum was normalized to its own mean and standard deviation. Smoothing was performed using a Savitsky–Golay [45] filter with a window size of 11 and a polynomial order of 2. As a result, preprocessed spectra formed a matrix ***X*** of size 191 × 855, where 191 is a number of samples, and 855 is a number of spectral wavelengths from the selected range called spectral features or just features. The obtained spectra were randomly divided into training and test samples in a ratio of 50:50. The training part was augmented with Gaussian noise [45].

To characterize and compare the groups of spectra, we used the concept of “spectral archetype” proposed in [53]. Briefly, a “spectral archetype” is an “ideal” spectrum that corresponds to an “ideal” analyte, free from random features arising from uncontrollable causes. To construct a “spectral archetype”, groups of spectra were brought to the same scale using normalization and averaging. For each spectral point, the standard deviation was calculated for the entire set of spectra of the group. The standard deviation was calculated for each Raman shift of the spectra. The procedure was performed using a built-in function in the software environment OriginLab (OriginLab Corp., Northampton, MA, USA). The “spectral archetype” was represented graphically as an average spectrum with its standard deviation. Thus, the “spectral archetype” simultaneously characterizes the spectrum itself and the width of the standard deviation describes the reproducibility.

### 2.6. ACE Classification with LDA

To separate the spectra of different ACEs, we used LDA. The result of this analysis is the transformation of the original space of spectral bands into a space of smaller dimension, in which the groups under study are well separable by the means of discriminant functions (*LD(**X**))* defined as:

(1) $$S_{W}=\overset{c}{\underset{i=0}{\sum }}\left(x-\bar{x_{i}}\right){\left(x-\bar{x_{i}}\right)}^{T}$$

(2) $$S_{B}=\overset{c}{\underset{i=0}{\sum }}\left(\bar{x_{i}}-\bar{X}\right){\left(\bar{x_{i}}-\bar{X}\right)}^{T}$$

(3) $$\bar{x_{i}}=\frac{1}{N_{i}}\sum_{x\in D_{i}}x_{k},$$

where *c* is the number of ACE groups, $x_{k}$ is the *k*−th row in ***X*** and $D_{i}$—set of spectra $x_{k}$ from one group. Matrices $S_{W},\;S_{B}$ are called Within-class scatter matrix and Between-class scatter matrix. Then the eigenvectors of the following matrix ***A*** were used to construct *LD(**X**)*:

(4) $$A=S_{W}^{-1}S_{B}$$

(5) $$V=\left[\vec{v_{max\lambda_{1}}},\vec{v_{max\lambda_{2}}}..\vec{v_{max\lambda_{k}}}\right],$$

where ***V*** consists of eigenvectors of matrix ***A*** (or components), corresponding to eigenvalues in descending order:

(6) Z=LD(X)=XV.

The algorithm was trained on preprocessed data using a maximum number of components equal to 2, which is the same as the first two columns of ***V***. So, the resulting matrix ***Z*** will have two columns, which correspond to the *x,y* coordinates of the spectrum or the values of functions *LD1(**X**)*, *LD2(**X**)*. Preprocessing procedures and data analysis were implemented within the framework of Python library Scikit-learn [54].

## 3. Results and Discussion

### 3.1. SERS Substrate Characterization

The SERS substrates technology, as well as their morphological, optical, electrophysical, and enhancement properties were described in detail in [4,17]. Briefly, X-ray photoelectron spectroscopy data (XPS) showed compositional identity to pure metallic silver [17,55]. The XPS spectrum of silver film is shown in Supplementary Figure S3. This was confirmed by an assessment of the electrophysical properties of the substrate, which showed that the films were not porous, since the dependence of the current on the applied potential was linear and did not correspond to the concept of hopping conductivity characteristic of porous films or films at or below the percolation threshold. At the same time, the determination of the permittivity by analyzing the spectra ellipsometric angles using the Drude–Lorentz equation showed a difference from the results in [56] for continuous opaque thin silver films, which can be caused by the influence of the surface nanostructure determined by the roughness parameters. To determine them, the atomic force microscopy (AFM) method was used, which showed the polycrystalline structure of the substrates. An AFM image of silver substrate is shown in Supplementary in Figure S4. The values of the roughness parameters showed that the surface can be defined as smooth on the macroscale, since the root-mean-square roughness parameter did not exceed 2 nm. However, on the microscale, we used the parameter R3z from Standard ISO 4287–1997 describing the vertical distance between the third highest peak and the third lowest valley. R3z was already 7.78 nm, which made it possible to consider local irregularities as hot spots that implement SERS [4]. As a result of the combination of these parameters, SERS is realized due to the nanostructured surface of the substrate.

The stability of the substrates was also assessed and follows from the constancy of the ellipsometric parameters, since the ellipsometric is highly sensitive to minimal changes in morphology and optical properties caused by degradation processes. The spectra of ellipsometric parameters were measured at three points of a freshly formed sample and after two months of storage. The spectra were in good agreement. The continuity of the films and the thickness of 100 nm determine the presence of good thermal conductivity. Moreover, we could see that ACEs did not degrade during the measurement of the spectra, despite the long accumulation time and the increased laser excitation power. This was shown by sequentially measuring the ACE spectra from one point 10 times consecutively without interruption. It appeared that the standard deviation of this measurement cycle was small (no more than 9%). The reproducibility of the spectra was also shown when measured at different points in the sediment. Sufficient reproducibility is confirmed by the value of the standard deviation, which did not exceed 15%. Evaluation of the enhancement properties of the substrate was provided based on the comparison of the Raman spectrum on glass and the SERS spectrum, measured under the same conditions (Figure S5). The enhancement coefficient of the substrate was determined as (1–1.5) × 10^3^ from the ratio of the amplitudes of the maximal vibrational band of the SERS spectrum and the Raman spectrum. Thus, the enhancement coefficient was relatively low, but we had enough opportunities for long-term accumulation of spectra at sufficiently high laser powers. The Raman spectra were characterized by a very low signal-to-noise ratio which made them unsuitable for comparative analysis of spectra of ACE produced by different cells. We could note, however, that the main vibrational bands in the Raman and SERS spectra of each separate ACE coincided.

In addition, as we used ACE in the presence of zinc salt necessary for maintaining active ACE conformation, we measured the SERS spectra of this salt alone. It appeared that zinc chloride showed two bands at 299 and 392 cm^−1^ (not shown) which did not affect the ACE spectra.

### 3.2. ACE SERS Measurement

**Figure 1:**
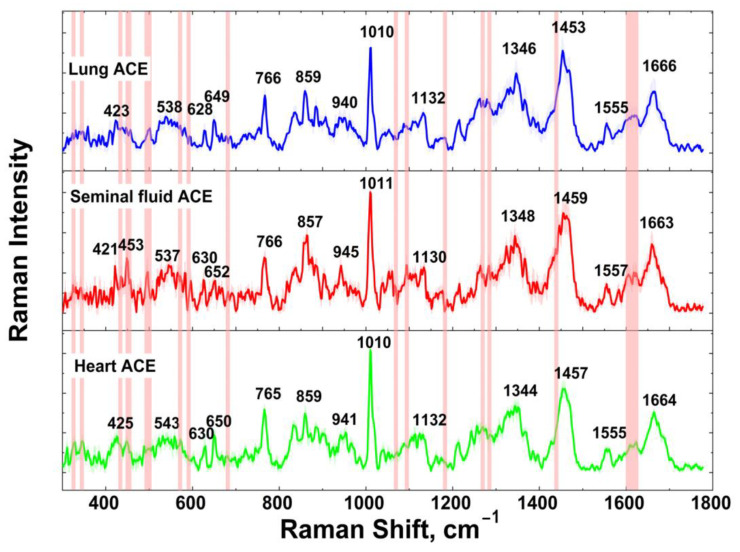
SERS spectra of ACEs. Red traces indicate those vibrational bands which contain the differences in ACE spectra.

Three types of ACE from seminal fluid, lung and heart tissues were purified and brought to a concentration similar to 1 μM. The SERS spectrum of each ACE represents a rich spectral picture (Figure 1).

Visually, the spectra were very similar and the positions of the main vibration bands were close or almost the same. Differences were noticeable in minor vibration bands. The positions of these bands in Figure 1 are marked with red traces. Note that we could not expect great differences between the spectra, as the protein structure of lung, heart and seminal fluid ACEs is the same. So, we could expect that the minor differences could be caused by different glycosylation of the ACEs from different sources.

The measurements were carried out in a coffering at an equidistant distance from the edges along the dash-dotted line as shown on optical image of coffering in Supplementary Figure S1. The exact mapping of the bands is shown in Table 1.

Many peaks are attributed mainly to the side-chain vibrations, but the main chain (***amide I*** and ***amide III***) is also presented in Table 1. In the SERS spectra, skeletal deformation of amino acids can be found below 450 cm^−1^ [61,70,71], thus, vibrations between 324–448 cm^−1^ are attributed to skeletal deformational modes. For band description in the spectra of three ACEs, we accepted the following order—“seminal fluid—lung—heart” ACE—and used this order further throughout the whole text. Thus, the most intense bands in the ACE spectra were observed at 766-766-765, 1011-1010-1010, 1348-1346-1344, 1459-1453-1457, and 1663-1666-1664 cm^−1^. These bands correspond to ***Met*** CH_2_ rocking, ***Trp*** indole ring breathing, ***Gly*** vibrations, ***Gly*** CH_2_ scissoring and ***Amide I***, respectively. Generally, SERS spectra of the same protein obtained by different research groups may vary because of selective enhancement of amino acids near a metal surface [59,61,70,72], which can lead to minor differences in the position of the determined vibration bands relative to the literature data. Some bands in ACE SERS spectra demonstrate higher enhancement than others due to many reasons. For example, ***Gly*** and amino acids adjacent to ***Gly*** are able to orient closer to the surface than amino acids with a bigger side chain. ***Met*** provides a strong signal because of sulfur atom attraction to the silver surface. ***Trp*** also has intensive SERS bands due to the strong conjugation of the indole ring with the substrate. In addition to ***Trp***, other aromatic amino acids make many contributions to the spectra: 652-649-650 ***His***, 766-766-765 ***Tyr***, and 1212-1215-1213 ***Phe***, due to similar mechanism.

The ACE molecule contains 142 ***Leu*** residues, ***Leu*** being the most abundant amino acid in the enzyme. Its content is almost 20% higher than for average protein [63], so, ***Leu*** may appear in SERS spectra of ACE more often than for average protein. We see contributions from ***Leu*** at 683, 963-963-966, 1241-1240-1243, (no)-1270-1270, 1324-1326-1327. These vibration bands are not intense, since ***Leu*** is a hydrophobic molecule and is not characterized by strong interactions with the substrate surface. Most likely, we can observe ***Leu*** fluctuations due to its proximity to other active amino acids.

We have shown earlier that the amino acid sequence, 1173–1203, located on the C terminus of seminal fluid ACE was in contact with silver substrate [17]. In the spectra of the three ACEs, we observed traces of the deformation vibrational band of the carboxylic group COO^−^ of ***Arg***1203 at 537-538-543 cm^−1^, which is the C-terminal residue. Since the silver film surface is characterized by a positive charge in water [17,63] we also see many vibration bands from negatively charged ***Glu*** and ***Asp***, as well as many vibration bands of aromatic groups in ***His*** and ***Phe***, characterized by an excess of electron density due to π-electrons. 

The adsorption of ACE molecules on the silver surface caused by the interaction of sulfur in amino acids and the silver surface led to the appearance of (*C-S*) band at 630-328-630 cm^−1^ in ***Met*** [64]. It is worth noting that we observed ***Met*** vibration bands in ACE spectra but did not observe bands corresponding to sulfur-containing ***Cys***. So, it can be assumed that ACE globules were oriented by the ***Met***-enriched regions towards the substrate surface. Since the content of ***Met*** is low (about 2.5%), it is easy to determine the limited areas of its accumulation, while the areas containing ***Cys*** turn out to be remote from the substrate.

However, some neutral and positively charged amino acid residues were traced in the spectra, which may be due to their spatial orientation and proximity to the silver surface, regardless of the nature of the side chain. The possibility of observing vibration bands of neutral and positively charged amino acid is due to the following. Interaction between the molecule and the silver substrate is provided due to COO^−^ group, S-containing amino acids, and aromatic groups. As a result of this, hydrophobic regions can also approach the substrate. It is shown from the review [73], based on a number of studies, that the region of the electromagnetic SERS mechanism extends to several nanometers far from the substrate surface. Consequently, the hydrophobic regions fall into the enhanced field region and can be enhanced, whereupon we also observe their vibration bands in our spectra. 

In addition to bands from single amino acid residues, bands of dipeptide vibrations were found in the spectra. To determine the bands of dipeptides, the literature data [61] devoted to the interpretation of the SERS spectra of a number of dipeptides were used. So, the position of these dipeptides in the amino acid sequence was determined with a sufficiently high accuracy. The most intensive characteristic peptide bands are 954-950-953 cm^−1^ (corresponding to dipeptides 589GE590, 1187GE1188), 1011-1010-1010 (representing 580WL581, 1178WL1179), and 1459-1453-1457 cm^−1^ (corresponding to dipeptide 1190LG1191).

Note that the spectra of lung and heart ACE contained vibrational bands at 1183 cm^−1^ corresponding to dipeptides ***Leu-Gly*** or ***Glu-Gly***, and at 1270 cm^−1^ corresponding to ***Ser*-*Gly*** or ***Leu-Glu***. Since these bands were observed only in lung and heart ACE spectra, these peptides were likely located in the stalk region and transmembrane segment absent in seminal fluid ACE which represents a soluble ACE form. So, these regions can be characteristic for the membrane form of ACE, which is often present as a contaminant in purified preparations of tissue ACE [74,75,76]. Thus, we can suggest the exact positions of dipeptides as follows, 1217LG1218, 1236LG1237, 1245LG1246, 1205EG1206 and 1211SG1212. 

The spectra of all three ACEs demonstrated the vibrational band at 963-963-966 cm^−1^, corresponding to the combination ***Pro-Leu*** localized on the substrate [17]. This combination is located only in the linker region between N and C domains in ACE structure and corresponds to 602PL603. The observation of the band from the linker region and, in general, the preservation of the shape of the spectra and the ratio of the amplitudes of the main bands suggest that tissue ACEs (from lung and heart) are characterized by the same model of landing on the silver SERS substrate, as was previously proposed for seminal fluid ACE [17]. In this model, the ACE globule is located on the substrate in such a way that some potential N-glycosylation sites ***Asn***9, ***Asn***25, ***Asn***82, ***Asn***648, ***Asn***666, ***Asn***731, and ***Asn***913 are far from the substrate surface [17]. In addition, the ***Asn***454 is located inside the protein globule and cannot interact with the substrate.

Of particular interest to us are potential N-glycosylation sites on which glycans could be in direct contact with the substrate, namely, ***Asn***131, ***Asn***289, ***Asn***416, ***Asn***685, ***Asn***1162, and ***Asn***1196. However, although the last two residues are in direct contact with the substrate in a proposed model [17], they are most likely not glycosylated [32,34,36]. Other N-glycosylation sites, ***Asn***45, ***Asn***117 and ***Asn***480, on the ACE globule are located at a short distance from the substrate and, therefore, glycans able to occupy an area of 200 Å or more [77] can also interact with the substrate and/or interfere with protein core–silver substrate interaction.

Thus, the presence or absence of glycans at potential N-glycosylation sites ***Asn***45, ***Asn***117, ***Asn***131, ***Asn***289, ***Asn***416, ***Asn***480 and ***Asn***685, as well as the fine structure of these glycans, could lead to the differences in the SERS spectra of ACE from different sources. As ACE from different sources could differ both in the position of really occupied glycosylation sites and in the glycan structures [32,34,37,78], the observed differences in the SERS spectra should be mainly associated with the appearance of vibration bands of different oligosaccharides, especially with the same landing of molecules on the substrate surface. Knowing that a small number of potential N-glycosylation sites are located near the substrate, we can assume that the differences in the spectra could not be significant. Indeed, the spectra were very similar, as can be seen in Figure 1, with the exception of some bands. Thus, the spectrum of seminal fluid ACE contained exclusive bands at 453, 492, 572, 591, 683, 1069, 1095 cm^−1^, which were absent in the lung and heart ACE spectra. Additionally, we could see the shifts in the maxima of the vibrational bands and a change in the shape of the peaks throughout the spectra of all types of ACEs.

Pairwise comparison of the heart ACE–lung ACE pair spectra shows that there is a band at 1623 cm^−1^ (indole NH in ***Trp(Trp-Leu)***) characteristic only for the lung ACE, while the bands at 502 (unknown band), 1279 (CH_2_ wag. in ***Trp(Trp-Leu***))), and 1600 cm^−1^ (ring C-C str. in ***Phe***) were characteristic only for the heart ACE. Similarly, pairwise comparison of the spectra in the seminal fluid ACE–lung ACE pair shows that the bands at 502 (unknown band), 1183 (in ***Glu-Gly, Leu-Gly***), 1270 (CH_2_ wag. in ***Trp(Trp-Leu)***), and 1613 cm^−1^ (sym. ring C-C in ***Gly(Tyr*-*Gly)***) were characteristic only for the lung ACE, while the bands at 572 (COO^−^ rock. in ***Thr***), 591 (NH def. in ***Trp***), 683 (C-S str. in ***Met***), 1069 (C-N str. in ***Pro(Pro-Leu)***), 1095 (C_α_-C-N str. asymm. in ***Pro-Pro***, and NH_2_ twist. in ***Met-Leu***), 1441 (CH_2_ sciss. in ***Gly(Leu-Gly)***), 1618 cm^−1^ (indole NH in ***Trp(Trp-Leu)***) were characteristic only for ACE from seminal fluid. These differences correspond to the position of the oscillation bands at the maximum and, most likely, are caused by the influence of adjacent glycan structures within different ACEs. However, a more precise analysis of the spectra could be carried out by the LDA described below.

### 3.3. ACE Classification with LDA

As ACE spectra differed subtly, these differences had to be considered in a complex way using machine learning methods. This approach might take into account even tiny differences.

**Figure 2:**
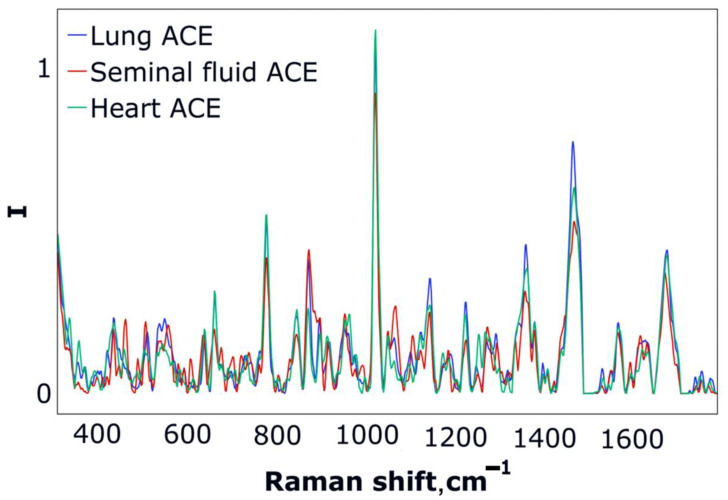
The averages of the collected spectra for each type of ACE from the selected range after baseline correction, normalization and smoothing.

The averages of the processed spectra for each ACE type are shown in Figure 2 and represent the images of the main groups of spectra, which will be used for calculation.

**Figure 3:**
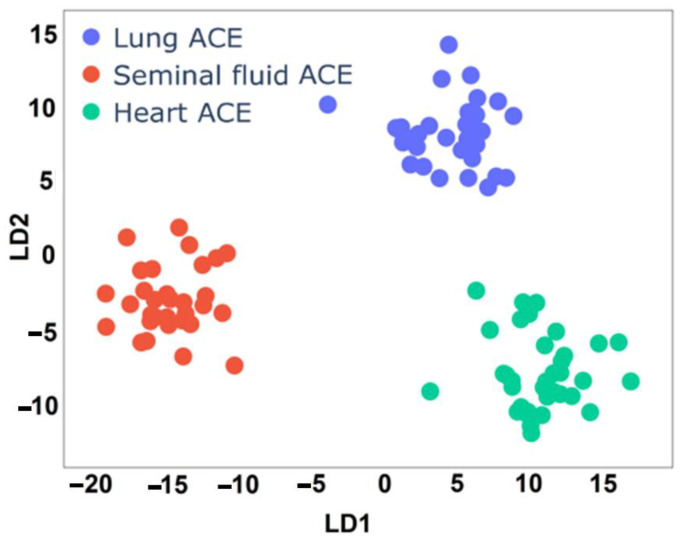
LDA subspace from test set defined by discriminant functions (matrix ***Z***). The corresponding protein groups from the test part are indicated by color: blue—lung ACE; red—seminal fluid ACE; green—heart ACE.

On the graph, the changes in the averaged spectra are represented over an entire frequency range. We could preliminarily visually estimate the expected changes from the bands (Figure 1). Although, with the help of LDA, we can determine two discriminative functions—*LD1(**X**)* and *LD2(**X**)*—which are a linear combination of all spectral features (***X***—spectral features, corresponding to each sample). The resulting subspace of LD functions is shown in Figure 3.

Figure 3 demonstrates the projections of the spectra on LDA axes 1 and 2, which approximate the original representation of the spectral array in 2D space. The spectra presented in this space define three groups of clouds that correspond to different ACE from definite source. The possibility of constructing separate disjoint clouds is a direct proof that the spectra of ACEs from different sources differ from each other (when projected on some axes in a smaller space) and can be separated using classification methods (linear classifier) as depicted in Table 2.

Table 2 shows the result of ACE separation, taking into account the entire study range on 99 deposited (test) samples. The accuracy of the prediction attains a value of 100%, which indicates that all samples were determined correctly.

### 3.4. Analysis

We evaluated the contribution of spectral bands (features) to the separation of ACE spectra using a linear classifier. To do this, the extra lanes were eliminated as follows:

- Contribution value (importance) to the separation exceeds 95% quantile in absolute value;
- Contributions at the boundaries of the studied range were not taken into account in the analysis.

**Figure 4:**
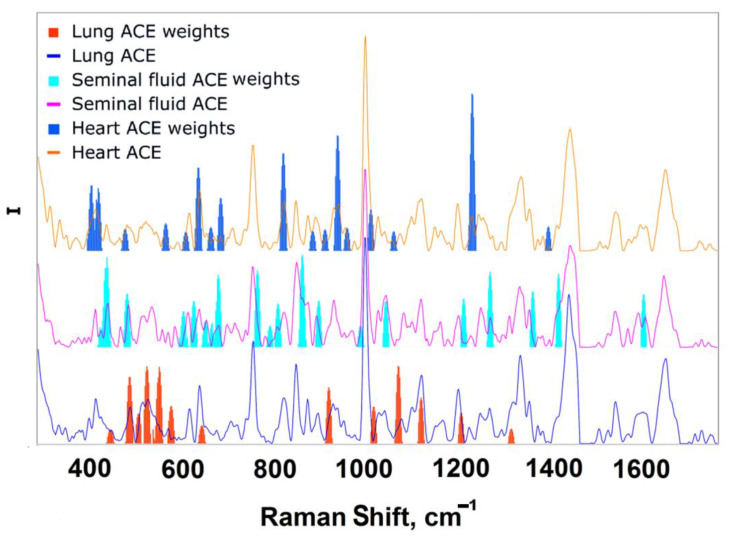
Feature importance analysis of trained LDA model. Bar plots indicate the most significant model weights for each ACE group after filtering described above. The average spectra of the corresponding ACE are also shown in the figure.

This approach helped to filter out some of the signs (~700) that included noise. The presented values were normalized to the maximum importance of contributions and are shown in Figure 4.

Figure 4 shows which bands have the greatest contribution to the separation of different ACEs. To verify this, the following experiment was carried out:

1. The values of the bar plot were sorted in descending order;
2. The first band was taken and the forecast was based on it;
3. The mean value of the target metric with its standard deviation on random train and test sampling from ***X*** was recorded;
4. The process continued until convergence.

**Figure 5:**
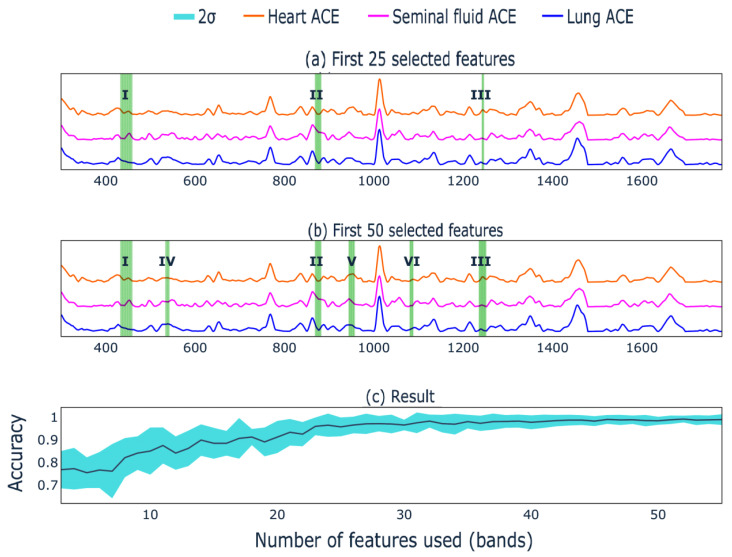
Feature contribution to the mean ACE spectra: (**a**) for first 25 selected features; (**b**) for first 50 selected features; (**c**) achieved accuracy with its confidence (2σ) interval vs number of selected features for the classification.

The resulting dependence is depicted in Figure 5, which demonstrates the position of the first 25 features forming three intervals (a) and the first 50 features forming six intervals (b). Three groups of green lines are seen in Figure 5a and six groups of green lines in Figure 5b. Figure 5c shows how many features are needed for maximum accuracy (equal to 1). Thus, the spectra of ACEs from three different sources can be successfully separated based only on three selected band intervals according to 25 first features. Further addition of bands to the calculation did not lead to any significant improvement in the result, since the accuracy takes values very close to 1 and its confidence interval is much smaller than at the beginning of the numerical experiment (Figure 5c).

**Figure 6:**
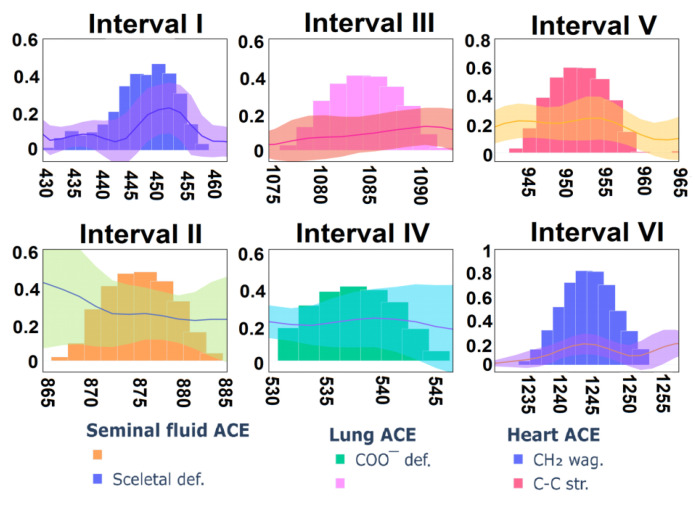
Selected intervals from Figure 5, assigned for each type of ACE, respectively. Bar plots correspond to the feature importance. Lines correspond to spectra averages for each ACE type.

It is important to show which vibration bands from preceding defined intervals made the greatest contribution to the differences between the spectra. The analysis based on the visualized contributions of the features made it possible to determine these bands. They are shown in Figure 6. Additionally, the corresponding spectra averaged over the group and their confidence interval were depicted in each subfigure. For ACE from seminal fluid, such intervals were: 431–458 cm^−1^ with feature importance 0.48, which corresponds to the band 435 cm^−1^ (skeletal def.), and 867-885 cm^−1^ with importance 0.47. For the heart ACE, such intervals were: 1235-1254 cm^−1^ with importance 0.81, which corresponds to the oscillation band 1243 cm^−1^ (CH_2_ wag. In ***Leu(Leu-Gly)***), and 944–960 cm^−1^ with importance 0.6, which corresponds to the oscillation band 953 cm^−1^ (C-C str. In ***Gly(Gly-Glu)***). For the lung ACE, such intervals were: 532–545 cm^−1^ with feature importance 0.4, which corresponds to the band 538 cm^−1^ (COO^−^ def. in ***Arg***), and 1077-1091 with importance 0.39. Note that these intervals do not match the differences between ACEs from Table 1, since the table reflects the vibrational band maximum position, while the band width and shape are taken into account by the LDA in the cumulative form of weights (Figure 4). Thus, the intervals reflect the most significant contributions (weights). It is seen that some of these intervals unambiguously correlate with the vibrational bands of the spectra of ACE from different sources. However, some, such as the differences in the intensity of the bands (Table 1), are most likely associated with the structure of glycans. Glycan fluctuations are unidentified due to their complexity and ambiguity, but they make the main contribution to the differences in ACEs spectra from different sources.

Previously, we have shown that lung ACE produced by lung endothelial cells, heart ACE, produced by heart endothelial and myofibrolast cells, and seminal fluid ACE, produced by epithelial cells of prostate and epididymis, can be differently glycosylated [32,34]. The most striking differences were found in the putative glycan structures on the ***Asn***666 glycosylation site; seminal fluid ACE and heart ACEs could contain complex and hybrid type glycans, respectively, while in the lung ACE, this site most likely bears the high mannose type glycan [34]. In the ACE model on the silver substrate, however, this site is located far from the substrate. We can especially select two from a list of potential N-glycosylation sites which are located near the silver substrate on the ACE model, namely, ***Asn***117 on the N domain of ACE and ***Asn***685 on the C domain. It was shown earlier by mass spectrometry of tryptic hydrolyzates of purified ACEs that these two ***Asn*** residues could be glycosylated in all three ACEs, i.e., from seminal fluid, lung and heart. The fine structures of glycans at these sites, e.g., the content of fucose and neuraminic acid, the number of branches, etc., may differ significantly [34]. Different glycosylation of ***Asn***117 and ***Asn***685 in different ACEs was indirectly confirmed by the differences in binding of monoclonal antibodies, recognizing epitopes on the ACE surface, to these ACEs. Namely, it was shown that the local surface conformation of ACE from seminal fluid differs from that of lung ACE in the ***Asn***685 region [32,34], while the surface of heart ACE differs from lung ACE in the ***Asn***117 region [33,34]. Therefore, we assume that it is the differences in the glycan structures at ***Asn***117 and ***Asn***685 that give the differences in the SERS spectra of ACE from different tissues.

## 4. Conclusions

For the first time, SERS spectra of human ACE from heart, lung and seminal fluid were measured and compared with full assignment. The difference between three ACEs, produced by different cells in the organism, was demonstrated using a numerical method based on machine learning—the LDA method. It was shown that, for spectra separation, it is sufficient to use the first 25 features. However, more thorough data could be obtained using 50 features. Three main ranges of features contributing to the separation were identified for each of the three ACE species. Several frequency intervals did not correspond to assignment vibration bands and therefore may belong to the glycan structures within ACE glycoprotein which are responsible for the main contribution to the separation of ACE from different sources. Thus, on the example of ACE, we first demonstrated the prospects and opportunities of the SERS method for distinguishing isoforms of a glycoprotein produced in different cell types and differently glycosylated.

The possibility of the application of the LDA method was shown for defining the most significant vibrational bands in the spectra of differently glycosylated proteins having equal protein structure. This approach makes it possible to extract chemical spectral information only through statistically pure methods and to define protein species produced in different cells. The use of deep mathematical methods in relation to medicine would expand SERS diagnostic capabilities, making it possible to determine tissue-specific proteins at pathologies.

## Figures and Tables

**Table 1:** ACE band assignment.

Seminal Fluid	Lung	Heart	Band Assignment	Amino Acid or Dipeptide	Reported Band	Ref
324	-	328	-	-	-	-
344	-	346	-	-	-	-
421	423	425	Skeletal def.	-	-	-
435	-	-	Skeletal def.	-	-	-
445	-	448	-	-	-	-
453	-	-	Benzene ring def.	Trp	454	[57]
492	-	-	COO^−^ bend. + CH_2_	Gly	497, 496	[58,59]
-	502	-	-	-	-	-
537	538	543	COO^−^ def.	Arg	535	[60]
572	-	-	COO^−^ rock.	Thr	568	[61]
591	-	-	NH def.	Trp	595	[62]
630	628	630	C-S str.	Met	632	[63]
652	649	650	Imidazole ring breathing	His	657	[59]
683	-	-	C-S str.	Met-Leu	685	[64]
766	766	765	CH_2_ rock.	Met	765	[59]
833	835	833	Ring breathing mode and out-of-plane	Tyr	837	[65]
857	859	859	Ring def. Fermi resonance		857	[65]
885	885	886	C-N str. + C_β_-C_δ_ str.	Ala	885	[66]
905	906	902		Asp	902	[59]
945	940	941	C-C str.	His	948	[57]
954	950	953	C-C str.	Gly(Gly-Glu)	956	[67]
963	963	966	C-C str.	Pro-Leu	961	[67]
1011	1010	1010	Indole asym. ring breathing	Trp(Trp-Leu)	1011	[67]
1041	1043	1037	C-N str.	Pro(Pro-Leu)	1044	[67]
1053	1052	1051	C_α_-N str., C-N str.	Met-Leu, Ala-Ala	1056, 1050	[63,67]
1069	-	-	C-N str.	Glu(Gly-Glu)	1066	[67]
1095	-	-	C_α_-C-N str. asym., NH_2_ twist.	Pro-Pro, Met-Leu	1092, 1095	[63,68]
1130	1132	1132	N-H wag.	Lys	1142	[61]
1169	1174	1173	N-H wag.	His	1160	[61]
-	1183	1183	-	Glu-Gly, Leu-Gly	1194, 1174	[67]
1212	1215	1213	Ring def.	Phe	1214	[59]
1241	1240	1243	CH_2_ wag.	Leu(Leu-Gly)	1241	[67]
1260	1262	1256	Amide III	-	1264	[61]
-	1270	1270	CH_2_ wag.	Leu(Leu-Glu), Ser-Gly	1276, 1266	[67]
1283	1279	-	CH_2_ wag.	Trp(Trp-leu)	1283	[67]
1307	1307	1307	CH_2_ wag.	Glu(Gly-Glu)	1307	[67]
1324	1326	1327	C-NH_2_ str.	Met-Leu	1323	[67]
1339	1333	1335	C-H bend.	Asp	1336	[69]
1348	1346	1344	-	Glu	1346	[59]
1366	1366	1367	Indole vibration	Trp(Trp-Leu)	1363	[67]
1385	1385	1386	-	-	-	-
1392	1394	1396	COO^−^ sym. str.	Leu-Leu	1396	[67]
1441	-	-	CH_2_ sciss.	Gly(Leu-Gly)	1440	[67]
1459	1453	1457	CH_2_ sciss.	Gly(Leu-Gly)	1454	[61]
1473	1467	1468	C_γ_, C_δ_ bend.	Arg	1477	[59]
1557	1555	1555	C_β_-C_γ_ def.	Lys	1556	[70]
1605	1600	-	Ring C-C str.	Phe	1602	[61]
-	1613	1611	Sym.ring C-C str.	Gly(Tyr-Gly)	1613	[67]
1618	-	1623	Indole NH	Trp(Trp-Leu)	1621	[61]
1663	1666	1664	Amide I	-	1664	[61]

C_α_, C_β_, C_γ_, and C_ε_ refer to the 1st, 2nd, 3rd, and 5th carbon atoms of the terminal COO^−^ group, respectively; def.—deformational, str.—stretching, sciss.—scissoring, bend.—bending, wag.—wagging, rock.—rocking, twist.—twisting, sym.—symmetrical, asym.—asymmetrical.

**Table 2:** Main classification metrics for each ACE group on test set.

	Precision	Sensitivity	F score	Quantity
Lung ACE	1.00	1.00	1.00	33
Seminal fluid ACE	1.00	1.00	1.00	31
Heart ACE	1.00	1.00	1.00	35

## Data Availability

Not applicable.

## Supplementary Materials

The following supporting information can be downloaded at: https://www.mdpi.com/article/10.3390/biomedicines10061389/s1. Figure S1. Coffering of dried ACE drop. The dash-dot line shows the location of the points along which the spectra were measured; Figure S2. Outliers on ACEs spectra: (a) heart ACE; (b) lung ACE; (c) seminal fluid ACE. Outliers are marked with red circles; Figure S3. XPS spectrum of substrate; Figure S4. AFM image of silver substrate morphology; Figure S5. ACE RS in comparison with SERS spectra: (A) heart ACE; (B) lung ACE; (C) seminal fluid ACE. SERS spectra reduced by a factor 10^3^.
