# Supplementary material for: Human Angiotensin I-Converting Enzyme Produced by Different Cells: Classification of the SERS Spectra with Linear Discriminant Analysis
Source: Biomedicines. 2022 Jun 12;10(6):1389. doi: 10.3390/biomedicines10061389 (PMC9219671; doi:10.3390/biomedicines10061389)
Supplement: Supplementary file 1 [file biomedicines-10-01389-s001.zip › biomedicines-1747757-supplementary.pdf]

Supplementary material

1. Code

The data presented in this study are openly available in https://github.com/roberts2510/ACE_classificationat “data” folder (data.csv). Computer code is available in python notebook in the same repository.

2. Coffering

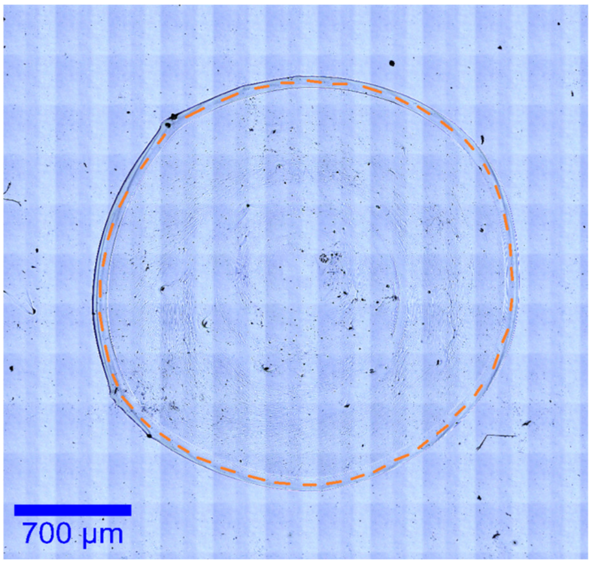

Figure S1. Coffering of dried ACE drop. The dash-dot line shows the location of the points along which the spectra were measured.

The figure shows optical typical image of a coffering of a certain width. The measurements were carried out at an equidistant distance from the ring edges along the dash-dotted line.

3. Outliers from the dataset

In the experimental data, the following outliers (red circles) caused by cosmic rays were observed and shown on the figure S2.

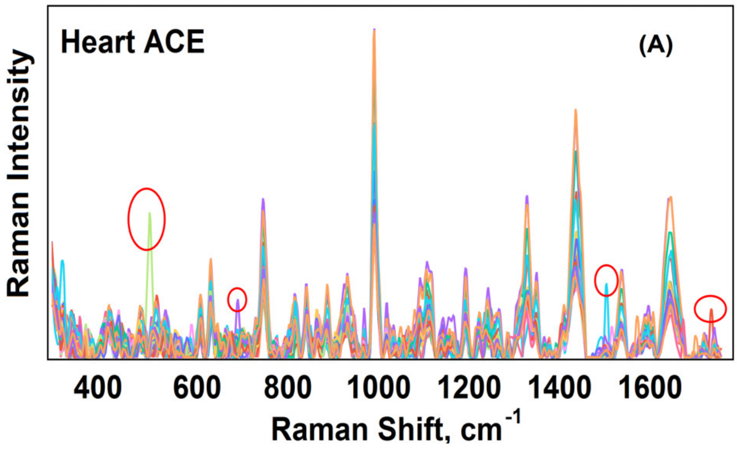

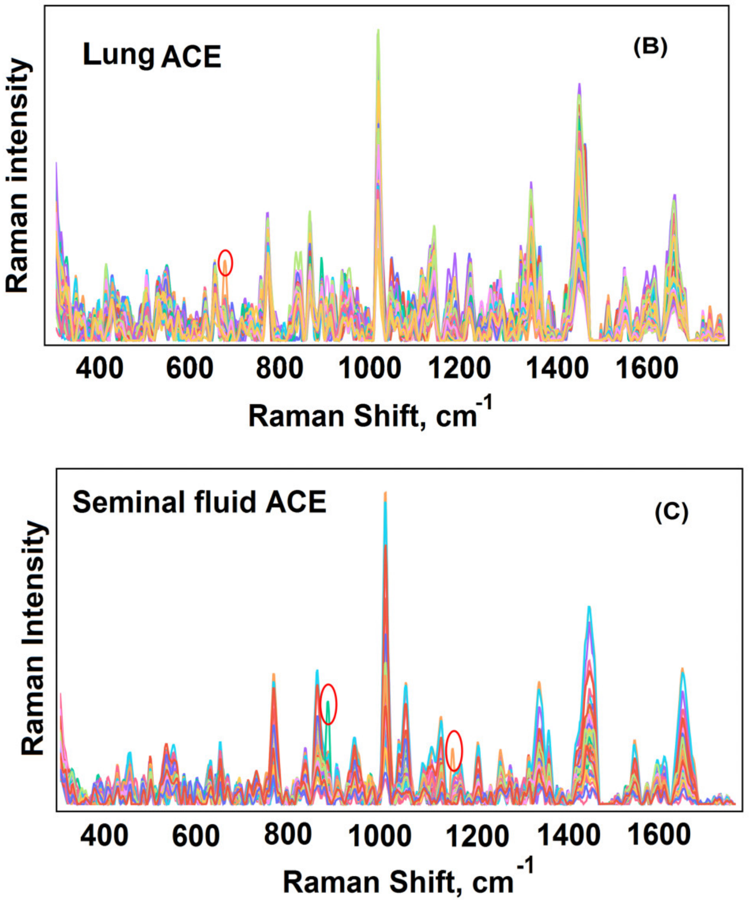

Figure S2. Outliers on ACEs spectra: (a) heart ACE; (b) lung ACE; (c) seminal fluid ACE. Outliers are marked with red circles.

Figure S2 shows the spectra for each ACE group: heart, lung and seminal fluid. Red circles indicate outliers caused by cosmic ray. Since the cosmic rays are an invariable attribute of vibrational spectroscopy, we had to use a reliable method to remove them. A distinctive feature is a high signal-to-noise ratio and a significant excess of intensity for vibration bands located at the same frequency. The criterion for their removal was the following:

$$\boldsymbol{I^{i}_{cosmic}} > \boldsymbol{I^{i}_{mean}} \qquad (S1)$$
$$on\ i - th\ band$$

Therefore, according to the criterion given above, they can be determined and removed manually or automatically.

4. **XPS spectrum**

XPS spectrum of silver substrate is shown in Figure S3.

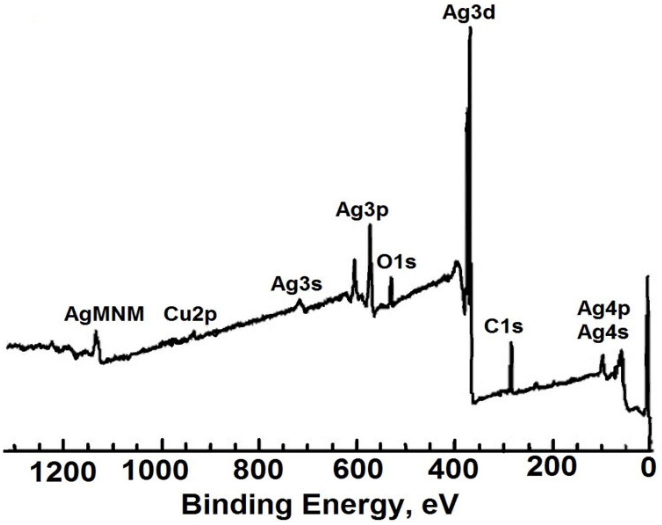

Figure S3. XPS spectrum of substrate

The spectrum shows the vibration bands of silver in the SERS substrate, as well as minor traces of oxygen, carbon and copper presented in the substrate.

5. **AFM substrate surface morphology**

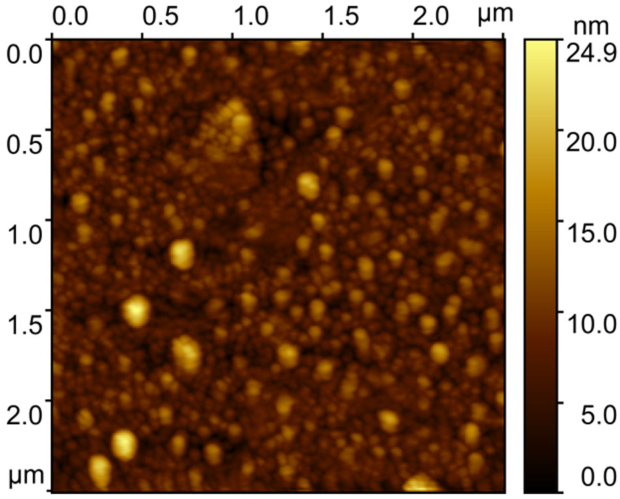

Figure S4. AFM image of silver substrate morphology.

The figure S4 shows the typical surface morphology of the substrates we use, obtained using an atomic force microscope in a tapping mode. The surface has a polycrystalline structure with a weakly pronounced roughness with separate heights and valleys responsible for the realization of the SERS effect. The color scale on the right shows the heights.

6. **ACE Raman spectra**

The ACE Raman spectra (RS) spectra were measured on glass substrates in the same way and with the same parameters as the SERS spectra. ACE RS are shown in figure S5 in comparison with SERS spectra.

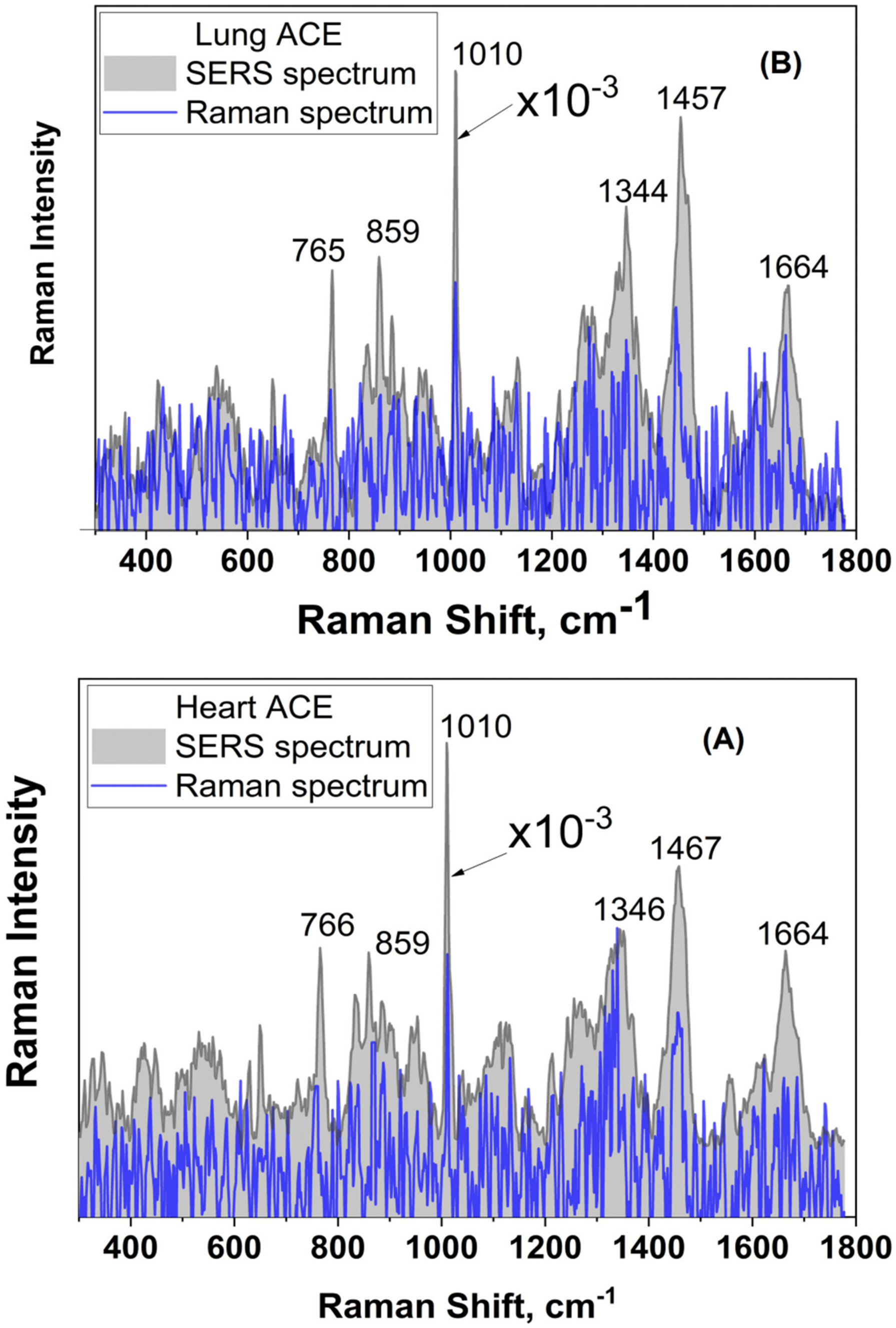

Lung ACE
SERS spectrum
Raman spectrum
(B)
1010
x10-3
765
859
1344
1457
1664
Raman Intensity
400 600 800 1000 1200 1400 1600 1800
Raman Shift, cm-1
Heart ACE
SERS spectrum
Raman spectrum
(A)
1010
x10-3
766
859
1346
1467
1664
Raman Intensity
400 600 800 1000 1200 1400 1600 1800
Raman Shift, cm-1

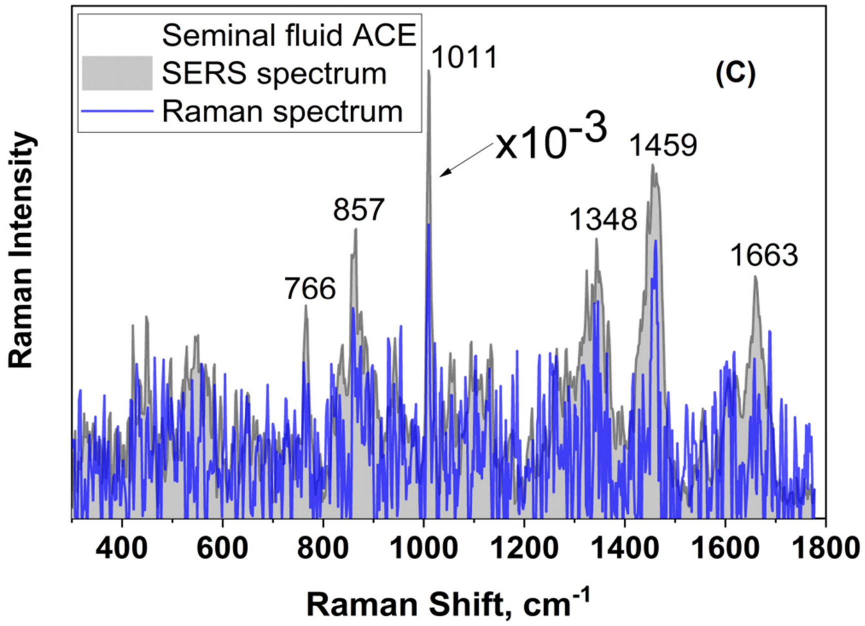

Figure S5. ACE RS in comparison with SERS spectra: (A) heart ACE; (B) lung ACE; (C) seminal fluid ACE. SERS spectra reduced by a factor 10^3.

To be able to compare the SERS and RS spectra, the SERS spectra were multiplied by a factor of 10^{-3}. It can be seen from the figure that RS are characterized by an extremely low signal-to-noise ratio, which made them unsuitable for comparative analysis of ACE from different tissues. We can only conclude that the positions of the main vibrational bands in SERS and RS spectra coincide.
